# Mice lacking neutral amino acid transporter B^0^AT1 (Slc6a19) have elevated levels of FGF21 and GLP-1 and improved glycaemic control

**DOI:** 10.1016/j.molmet.2015.02.003

**Published:** 2015-02-16

**Authors:** Yang Jiang, Adam J. Rose, Tjeerd P. Sijmonsma, Angelika Bröer, Anja Pfenninger, Stephan Herzig, Dieter Schmoll, Stefan Bröer

**Affiliations:** 1 Research School of Biology, The Australian National University, Canberra, ACT 0200, Australia; 2 Joint Research Division Molecular Metabolic Control, German Cancer Research Center, Center for Molecular Biology, Heidelberg University and Heidelberg University Hospital, 69120 Heidelberg, Germany; 3 Sanofi-Aventis Deutschland GmbH, Industriepark Hoechst, Frankfurt am Main 65926, Germany

**Keywords:** Type 2 diabetes, Epithelial transport, Amino acid metabolism, aWAT, abdominal white adipose tissue, BAT, brown adipose tissue, BM, body mass, IPGTT, intraperitoneal glucose tolerance test, IPITT, intraperitoneal insulin tolerance test, iWAT, inguinal white adipose tissue, NEFA, non-esterified fatty acids, RER, respiratory exchange ratio, WAT, white adipose tissue

## Abstract

**Objective:**

Type 2 diabetes arises from insulin resistance of peripheral tissues followed by dysfunction of β-cells in the pancreas due to metabolic stress. Both depletion and supplementation of neutral amino acids have been discussed as strategies to improve insulin sensitivity. Here we characterise mice lacking the intestinal and renal neutral amino acid transporter B^0^AT1 (*Slc6a19*) as a model to study the consequences of selective depletion of neutral amino acids.

**Methods:**

Metabolic tests, analysis of metabolite levels and signalling pathways were used to characterise mice lacking the intestinal and renal neutral amino acid transporter B^0^AT1 (*Slc6a19*).

**Results:**

Reduced uptake of neutral amino acids in the intestine and loss of neutral amino acids in the urine causes an overload of amino acids in the lumen of the intestine and reduced systemic amino acid availability. As a result, higher levels of glucagon-like peptide 1 (GLP-1) are produced by the intestine after a meal, while the liver releases the starvation hormone fibroblast growth factor 21 (FGF21). The combination of these hormones generates a metabolic phenotype that is characterised by efficient removal of glucose, particularly by the heart, reduced adipose tissue mass, browning of subcutaneous white adipose tissue, enhanced production of ketone bodies and reduced hepatic glucose output.

**Conclusions:**

Reduced neutral amino acid availability improves glycaemic control. The epithelial neutral amino acid transporter B^0^AT1 could be a suitable target to treat type 2 diabetes.

## 1 Introduction

In the development of insulin resistance, the role of protein nutrition in general, and of neutral amino acids in particular, is controversial. When provided in excess, amino acids, like fat and carbohydrate, can cause insulin resistance [1–3]. This is thought to involve the amino acid sensing mTORC1 complex [4,5]. Activation of mTORC1 and its downstream protein kinase p70S6K are known to phosphorylate insulin receptor substrate, which in turn will reduce the response to insulin [1]. Cellular, animal and human intervention studies, however, have yielded controversial results. Some studies reported improved insulin sensitivity after supplementation of leucine and other branched-chain amino acids [6–8], while other studies reported improved insulin sensitivity when neutral amino acids were curtailed [9,10]. A comprehensive feeding study in mice using different ratios of the three macronutrients pointed to a correlation between high protein intake, mTOR activity and morbidity at an advanced age [11]. Epidemiological studies have consistently shown that slight elevations of plasma neutral amino acids are good predictors of future diabetes [12,13]. Thus it has been proposed that elevated intake of these amino acids causes insulin resistance through a variety of mechanisms including mTOR activation and generation of short-chain fatty acids [14]. While abundance of amino acids is sensed through the mTOR pathway, depletion of amino acids is sensed through the GCN2/ATF4 pathway [15]. This pathway is activated by uncharged tRNA's, resulting in a general shutdown of protein translation through phosphorylation of protein translation initiation factors. At the same time selected transcripts are activated through an amino acid response element. Among the transcripts that are activated through this response are amino acid transporters in the pancreas [16] and FGF21 in the liver [17,18]. FGF21 is a metabolic hormone that induces a metabolic program adapting the body to fasting. Originally discovered as a hormone that increases glucose uptake into adipocytes independent of insulin [19], FGF21 generates a complex pattern of effects such as reduced serum glucose, triglycerides, fatty acids and insulin, while ketone body production is increased [19,20]. It is thought that most of the FGF21 metabolic effects are generated through its action on adipose tissue [21], where it also increases adiponectin release [22]. Amino acids also act as secretagogues of insulin secretion in β-cells or indirectly through the secretion of GLP-1 and GIP by L- and K-cells in the intestine [23,24]. Calorie-reduced high protein diets are frequently used as a tool to manage weight problems [25]. Low protein diets, by contrast, have been proposed to cause hyperphagic behaviour resulting in obesity [26]. Related to this amino acids are sensed by the central nervous system and play an important role in the generation of satiety [27].

Due to the multitude of effects that amino acids have in the regulation of metabolism, pharmacological targets have so far failed to emerge. While acute use of mTOR inhibitors indeed reduces insulin resistance, chronic use of mTOR inhibitors, given for instance as immunosuppressants after transplantation, can induce insulin resistance and cause diabetes [28]. Plasma amino acid levels are regulated through transport, metabolic processes and protein turnover. Liver, muscle, intestine, brain and kidney are organs with significant amino acid metabolism, while muscle is the main organ for storage [29]. Amino acid intake is mediated by group-specific amino acid transporters in the intestine, while the kidney filters and reabsorbs amino acids [30]. The amino acid transporter B^0^AT1 (*Slc6a19*) is the major epithelial transporter for neutral amino acids in the intestine and kidney [31]. As a result lack of Slc6a19 causes a selective deficiency of neutral amino acids, which can be used to address the role of neutral amino acids in metabolic regulation [32]. The deficiency is incomplete because amino acids are also absorbed as di- and tri-peptides via peptide transporter PepT1 in the intestine [33]. Inactivating mutations in B^0^AT1 (*SLC6A19*) in humans are known to cause Hartnup disorder, a largely benign disorder, which is characterised by large amounts of neutral amino acids spilling over into the urine [34–36]. To reach the plasma membrane B^0^AT1 requires coexpression of the ancillary protein collectrin (TMEM27) in the kidney [37] and angiotensin converting enzyme 2 (ACE2) in the intestine [38]. Here we show that lack of the neutral amino acid transporter B^0^AT1 improves glycaemic control through two opposing signals: first, an increased luminal load of amino acids in the intestine and, second, a reduced systemic amino acid availability.

## 2 Material and methods

### 2.1 Animal holding and high-fat diet

Mouse strain: *Slc6a19* (−/−) mice were used as described before [32]. Littermates were used as comparisons for all experiments. The number of animals used in each experiment is provided in the figure captions. Male mice were used for all physiological tests. Animal experiments were approved by the animal experimentation ethics committee of the Australian National University (R.BSB.06.10). Two months and six months old female mice were used for the high-fat diet experiment. Four mice were housed per cage. High fat diet food was custom designed and manufactured in dry, pelleted form by Specialty Feeds (By energy 46% fat, 21.6% protein, 32.4 carbohydrates). Animals were kept on the diet for 4 months; body weight was measured twice weekly.

### 2.2 Behavioural and metabolic phenotyping

Mice were individually housed for about 1wk prior to entering the PhenoMaster Cage System (TSE Systems, Bad Homburg, Germany), which enables simultaneous determination of indirect calorimetry, 3D activity, as well as food and water consumption. Mice were then housed individually in the system for a total of 8d. Calculations of total, lean- and body- mass adjusted oxygen consumption, carbon dioxide production rates and respiratory exchange ratio were performed according to established guidelines [39,40], averaged over the final 3d of housing, after a period of adaptation to the system. All mice were maintained on a 12 h light–dark cycle at 22 °C with unrestricted access to food and water. Upon exiting the system, body composition was determined by an Echo magnetic resonance imaging (ECHO-MRI) body composition analyser (Echo Medical Systems, Houston, USA). Faecal energy output was measured with the IKA C7000 calorimeter (IKA, Staufen, Germany) from an aliquot of lyophilised, pulverised faecal material collected over a 24 h period. Rectal temperature was measured using a sensor (N856-1) and digital measurement device (Almemo 2390-1; Ahlborn Mess-und Regelungstechnik GmbH, Germany), and were made between ZT3-5 on two separate days in a randomised order at standard laboratory temperature (i.e. 22–24 °C).

### 2.3 GTT, IPITT, pyruvate challenge, tissue glucose uptake

GTT: Mice were fasted overnight (16 h) before the test. Glucose was injected intraperitoneally (IPITT) or given by gavage (OGTT) (2 g/kg body weight) and blood samples were taken from the tail vein before and 30, 60, 90 and 120 min after the injection of glucose. Blood glucose was determined with an Accu-chek Performa glucometer. IPITT: To avoid hypoglycaemia, ITT were performed on randomly-fed mice during daytime. The mice were injected intraperitoneally with insulin (0.75 U/kg) in approx. 0.1 ml 0.9% NaCl. Blood samples were taken from the tail vein before and 15, 30, 45, and 60 min after the injection of insulin for the determination of blood glucose using an Accu-chek Performa glucometer. Pyruvate challenge: Mice were fasted for 4 h before the test. Pyruvate was injected IP (2 g/kg body weight) and blood samples were taken before and 15, 30, 60 and 120 min after the injection of pyruvate. Blood glucose was measured using Accu-Chek Performa glucometer.

Tissue glucose uptake: Mice were fasted for 16 h before intraperitoneal injection of 12μCi/mouse [2-^14^C]deoxyglucose ([2-^14^C]DG) together with glucose at a final concentration of 2 g/kg. After 40 min, blood glucose was measured using an Accu-Chek glucometer and a blood sample taken to determine the specific activity of glucose in the blood. Subsequently, mice were sacrificed and tissues were excised and rinsed in ice-cold PBS/1 mM EDTA pH 7.4. Pieces of about 50 mg were then snap-frozen in liquid nitrogen, weighed and homogenised in 0.5 ml 0.5% perchloric acid. After centrifugation in a table top centrifuge 0.4 ml of the supernatant was removed and neutralised with 0.4 ml 0.3N KOH. One aliquot of the homogenate was used without further treatment to measure the combined total activity of [2-^14^C]DG and [2-^14^C]deoxyglucose-6P ([2-^14^C]DGP) (Beckman LS 5000TD, Beckman Instruments, USA). A second aliquot of the homogenate was treated with 0.15 ml 0.3M Ba(OH)_2_ and 0.15 ml 0.3M ZnSO_4_ to precipitate [2-^14^C]DGP and was then counted to yield [2-^14^C]DG radioactivity. After adjusting for volume, the difference between the two readings, was used as the amount of [2-^14^C]DGP. Specific activity was calculated using blood [2-^14^C]DG volume activity in combination with blood glucose concentration.

### 2.4 Metabolomic analysis

**GC/MS:** To detect short-chain fatty acids in faeces a 20 mg sample was collected. The sample was mixed with 120 μl 0.2 M HCl, 300 μL diethyl ether and 24 μl of 50 mg/ml heptanoic acid as internal standard. The sample was extracted by vortexing at room temperature for 10 min. After centrifugation in a table-top centrifuge for 5 min at maximum speed, 50 μl of the organic layer was transferred to a new tube. For derivatisation 40 μl of 20 mg/mL Methoxylamine-HCl in pyridine was added and the sample incubated for 90 min at 30 °C. Subsequently 30 μl of N-tert-butyldimethylsilyl-N-methyltrifluoroacetamide (MTBSTFA)/1% t-butyldimethylchlorosilane (TBDMCS) was added and the sample was incubated at 80 °C for another 30 min. Derivatised metabolites were separated by gas chromatography using a Varian FactorFour Capillary Column (VF-5ms 30 m × 0.25 mm ID, DF = 0.25 μm + 10 m EZ-Guard column) on an Agilent 7890A GC system with the following parameters: Injection mode: Splitless; Inject volume: 1 μl; Inlet temperature: 220 °C; Carrier gas: Helium; Flow rate: 1 mL/min (7.6522psi); Temperature program: 50 °C for 1 min, then increasing by 5⁰C/min to 80 °C, (holding for 3 min) followed by an increase at 30 °C/min to 275 °C (holding for 5 min), resulting in a total run time of 21.5 min.

Compounds were detected by an Agilent 5975C MS using the following conditions: Ionisation: Electron impact (EI) @ 70 eV; MS acquisition modes: full scan (50–700 m/z, 4 scans/min) for non-targeted metabolites; selected ion monitoring (SIM) with ion dwell time of 40 ms for quantification of isovaleric acid; Transfer line temperature: 275 °C; MS source temperature: 250 °C; Quadrupole temperature: 150 °C; Solvent delay: 10 min.

**LC/MS/MS** was used to quantify amino acid concentrations in plasma and urine. Analysis, including sample preparation and derivatization was performed using the EZ:faast™ kit (Phenomenex, Torrance, USA) according to the user's manual. For LC/MS/MS detection, an electrospray ionisation-triple quadrupol mass spectrometer (Quantum Ultra, Thermo) coupled to a liquid chromatography system (UltiMate3000, Dionex) controlled by Xcalibur 2.0.7 software with Dionex Chrom MS link 6.80 was used. Chromatographic separation was achieved on an EZ:faast AAA-MS column 250 × 2 mm (Phenomenex) at 35 °C with a flow rate of 250 μL/min; autosampler temperature was set to 10 °C. A sample volume of 1 μl was injected onto the column. Eluents consisted of 10 mM ammonium formate in water (A) and 10 mM ammonium formate in methanol (B). Initial conditions (0 min) were 68% B, then linear gradient was applied within 13 min to 83% B. The system returned to initial conditions within 4 min and equilibrated for 7 min, resulting in a total run time of 23 min per sample. The column flow was directly converted into the H-electrospray ionisation (HESI) source of the mass spectrometer, which was operated in the positive ion mode. Capillary and vapouriser temperatures were maintained at 350 °C and 50 °C, respectively. Sheath gas and auxiliary gas were operated at 40 and 25 (pressure, arbitrary units), no ion sweep gas was applied. Collision energy and tube lens offset were adjusted accordingly to obtain the highest response for the specific amino acid. Quantification was performed via peak area ratios applied to internal standards provided in the kit.

### 2.5 Metabolite and hormone analysis

Serum levels of insulin, triglycerides, cholesterol, ketone bodies, non-esterified fatty acids (NEFAs), FGF-21, GLP-1, and GIP were determined using commercial assays as listed in Suppl. Table 1.

### 2.6 Western blots and antibodies

Frozen tissue samples were sectioned into 100 mg blocks and homogenised in 1 mL of T-PER Tissue Protein Extraction Reagent (Thermo Scientific). Protein was measured using the Bradford assay. Protein samples were separated by SDS-PAGE and blotted onto nitrocellulose membranes by electrophoretic transfer. After blocking nonspecific binding sites, proteins were probed using antibody dilutions as listed in Suppl. Table 2. Proteins were detected by enhanced chemiluminescence (ECL system, GE Healthcare). Blots were stripped and re-probed to detect proteins in phosphorylated and non-phosphorylated forms, and housekeeping genes as loading controls.

### 2.7 qRT-PCR

qRT-PCR was performed on a 7900HT Fast Real-Time PCR System (Applied Biosystems) with SYBR green and Platinum DNA polymerase (Invitrogen). The *GAPDH* gene was used as reference and relative transcript levels of biosynthesis were calculated with the ΔΔCt method.

### 2.8 Statistical analysis

The number of mice used for each experiment is stated in the figure legends. When comparing parametric data from two groups, the unpaired t test was used. *, p < 0.05; **, p < 0.01; ***, p < 0.001. CellProfiler software was used to analyse adipocyte area [41]. Statistical analyses of the calorimetric data were performed by SPSS, to normalise for differences in bodyweight or body composition data were analysed using ANCOVA, significant difference assumed at P < 0.05.

## 3 Results

### 3.1 *Slc6a19 (−/−) mice* have improved glycaemic control

**Figure 1:**
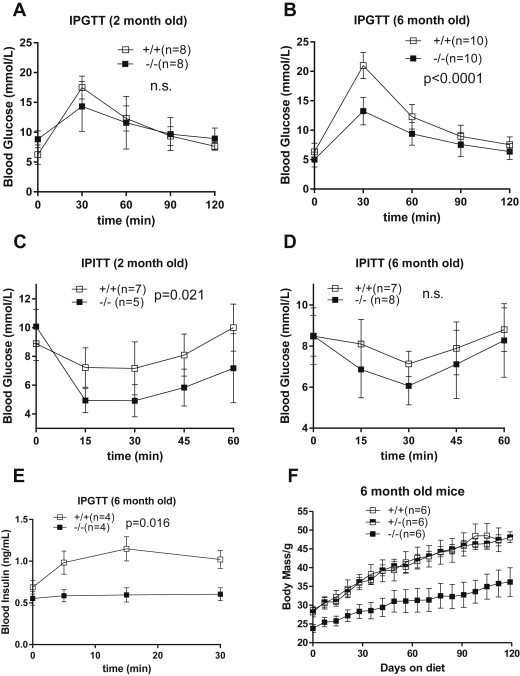
**Glucose tolerance and insulin tolerance of wildtype and Slc6a19(−/−) mice**. (A, B) Glucose (2 g/kg) was injected IP to measure glucose tolerance in 2 and 6 months old fasting mice. (C, D) Insulin (0.75 U/kg) was injected IP to measure insulin tolerance in randomly fed mice. (E) IPGTT was performed as in (A, B) but insulin levels were determined by ELISA. (F) Weight development on a high-fat diet. Statistical significance for AUC comparison between groups is shown in panels A–E. Female mice were used for the experiment in panel F; all other experiments were performed with male mice.

As we have shown previously, *Slc6a19 (−/−)* mice lack the major transport activity for neutral amino acids in both intestine and kidney. This causes reduced amino acid absorption in the small intestine and loss of amino acids through the urine ([32] and Figure S1). We also obtained preliminary evidence that upon feeding after fasting, insulin levels remained low although glucose levels were close to normal, suggesting that insulin sensitivity might be enhanced in *Slc6a19 (−/−)* mice [32]. To analyse glucose homoeostasis in more detail we performed glucose tolerance tests and insulin tolerance tests. Glucose tolerance was significantly improved in 6 months old mice (AUC, p < 0.0001), while the difference in 2 months old mice was non-significant (Figure 1A, B). Insulin sensitivity was improved in 2 months old mice (AUC, p = 0.021), but did not reach significance in 6 months old mice (Figure 1C, D). Surprisingly, very little insulin secretion was observed during an IPGTT in 6 months old mice (Figure 1E, note the different time scale, AUC p = 0.016). When placed on a high-fat diet, 6 months old *Slc6a19 (−/−)* mice gained less weight than aged-matched wildtype and heterozygous mice (Figure 1F). Glucose tolerance was similar in both groups of mice after 4 months on a high-fat diet, but the insulin response was more sensitive in *Slc6a19 (−/−)* mice (Figure S2).

**Figure 2:**
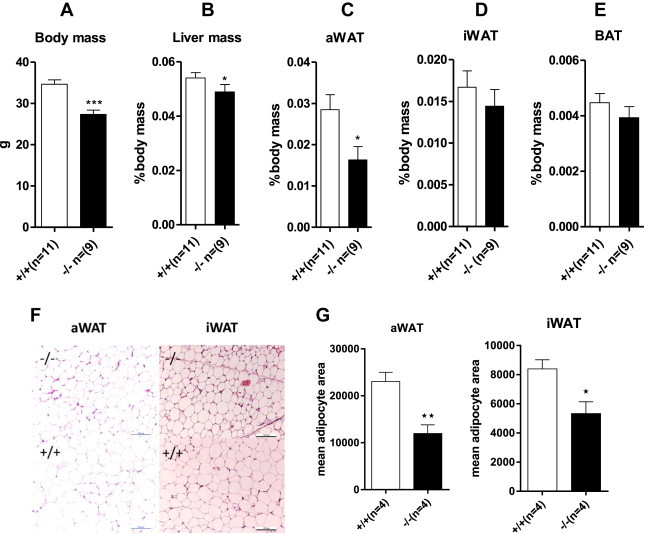
**Body composition of 6 months old wildtype and Slc6a19(−/−) mice**. Animal and organ masses were determined by weighing: body mass (A), liver mass (B), abdominal white adipose tissue (C), inguinal white adipose tissue, (D), and brown adipose tissue (E). Organ mass is displayed as % of body mass. (F) H&E staining of aWAT and iWAT adipocytes, scale bar is 100 μm. (G) Statistical analysis of adipocyte area in abdominal and inguinal white adipose tissue. Male mice were used for the experiments.

Analysis of body composition on normal chow showed a slightly reduced body weight and liver mass but more importantly a pronounced reduction of abdominal white adipose tissue (Figure 2A–C). Inguinal adipose tissue and brown adipose tissue were similar (Figure 2D, E). When normalised relative to body weight, only the abdominal white adipose tissue remained significantly reduced in *Slc6a19* (−/−) mice. Moreover, average adipocyte area in *Slc6a19* (−/−) mice was only half of that observed in wildtype mice (Figure 2F, G). When expressed as % body weight, the total body fat was similar in both groups: (+/+) Fat body mass 5.0 +/− 1.1 g (18.6%), lean body mass 20.3 +/− 1.5 g (75.5%), n = 11; (−/−); Fat body mass 3.7 +/− 0.7 g (16.4%), lean body mass 17.5 +/− 1.3 g (77.8%), n = 8.

### 3.2 *Slc6a19 (−/−) mice* are not calorie restricted

**Figure 3:**
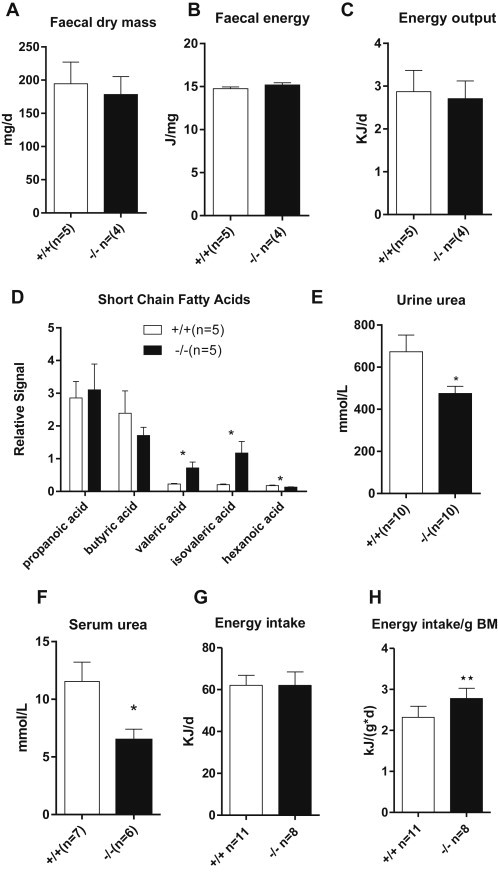
**Energy consumption in wildtype and Slc6a19(−/−) mice**. Faecal dry mass (A), faecal energy (B) and faecal energy output (C) was measured. (D) The amount of short-chain fatty acids in faecal samples was determined by GC–MS. Urea content was determined in randomly fed animals in urine (E) and serum (F). A Phenomaster system was used to determine daily food intake (G), and food intake per body mass (H). Male mice were used for the experiments.

A straight-forward explanation for the reduced weight gain on a high-fat diet would invoke reduced nutrient uptake in the intestine. It is important to note that reduced intestinal absorption will not explain the difference observed in the glucose or insulin tolerance tests because of the injection occurring into the peritoneum. The amount and energy content of the faeces was the same in both animal groups (Figure 3A–C), suggesting that amino acids get absorbed in the distal intestine and/or are converted by the intestinal microflora into fermentation products, which are absorbed by the colon. In support of this notion we observed increased amounts of valeric and isovaleric acid, which are fermentation products of branched-chain amino acids (Figure 3D). Fermentation products arising from complex carbohydrates (propionic acid, butyric acid), by contrast, were similar in both groups of mice. This indicates that amino acids are transferred to more distal sites of the intestine where they are fermented by the microflora. However, all nutrients are eventually absorbed leaving the same amount of energy behind in the faces.

To determine the impact of reduced absorption and loss of neutral amino acids in the urine we quantified amino acid levels in blood plasma and urine (Figure S1A, S1B). With the exception of 3-methylhistidine, plasma amino acid levels were found to be very similar, while urine neutral amino acid levels were highly elevated. These amino acid levels are consistent with those observed in human Hartnup disorder [42]. Two factors contributed to the stabilisation of plasma neutral amino acid levels in *Slc6a19 (−/−)* mice. First, muscle protein breakdown was reduced as indicated by lower plasma levels of 3-methylhistidine (Figure S1A), and, secondly, amino acids were spared as energy substrates as indicated by lower levels of urea in plasma and urine (Figure 3E, F). In summary, *Slc6a19 (−/−)* mice are not calorie restricted, but use fewer amino acids for energy generation and show reduced muscle protein turnover to keep plasma amino acid levels at close to normal levels. Despite gaining less weight on a high-fat diet, *Slc6a19 (−/−)* eat slightly more chow when normalised to body mass (Figure 3G, H).

### 3.3 *Slc6a19 (−/−) mice* have increased energy expenditure

**Figure 4:**
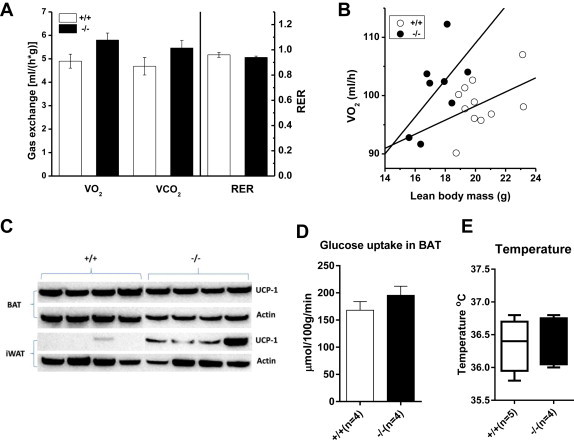
**Energy balance in wildtype and Slc6a19(−/−) mice**. A Phenomaster system was used to determine oxygen consumption, CO_2_ production and respiratory exchange ratio (RER) (A). To calculate body-weight independent energy usage, lean body mass was correlated with oxygen consumption (B). The relative amount of uncoupling protein (UCP-1) in brown adipose tissue (BAT) and inguinal white adipose tissue (iWAT) (C) was determined by Western blotting and immunodetection (D). Glucose uptake was determined by measuring [^14^C]DG uptake into BAT. Body temperature of both groups is shown in (E). Male mice were used for the experiments.

As an alternative, increased energy expenditure may explain the reduced weight gain on a high-fat diet. To investigate this possibility, we performed indirect calorimetry and analysed expression of uncoupling protein in brown adipose tissue. When normalised to lean body weight, oxygen consumption and CO_2_ production was elevated in *Slc6a19 (−/−)* mice, but the respiratory exchange ratio (RER) was very similar (Figure 4A). At a fixed hypothetical lean body weight of 19.1 g the data analysis predicted a VO_2_ of 104 ± 2 ml/h and 96 ± 2 ml/h for *Slc6a19 (−/−)* and wildtype, respectively (p = 0.033) (Figure 4B). This demonstrates increased energy expenditure in the *Slc6a19 (−/−)* mice independent of the known body-weight effect on energy consumption [43]. However, there was no increase of BAT volume in *Slc6a19 (−/−)* mice, and no increase of UCP1 expression in BAT (Figures 2 and 4C). Consistent with these data, BAT glucose consumption (Figure 4D) and body temperature (Figure 4E) was the same in both groups of mice. To explain the increased energy expenditure we wondered whether browning of white subcutaneous adipose tissue might occur in *Slc6a19 (−/−)* mice. In support of this notion we could detect significant expression of UCP-1 in inguinal WAT in *Slc6a19 (−/−)* mice, but not in wildtype littermates (Figure 4C).

### 3.4 *Slc6a19 (−/−) mice* show elevated levels of FGF21

**Figure 5:**
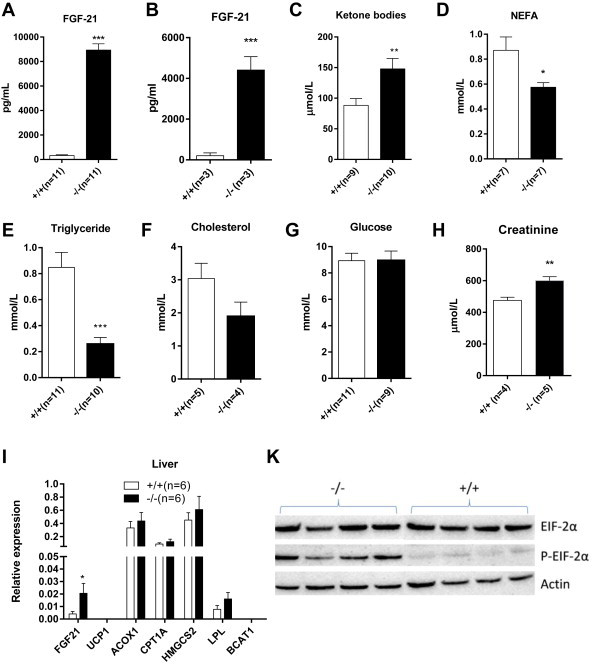
**Metabolite and gene expression profiles in wildtype and Slc6a19(−/−) mice**. Serum content of FGF-21 was measured by ELISA in 6 months old animals (A) and 2 month old animals (B). Metabolite levels were determined using enzymatic tests (C–H). Quantitative PCR was used to determine liver expression levels of fibroblast growth factor 21 (FGF-21), uncoupling protein 1 (UCP-1), fatty acid acyl oxidase-1 (ACOX-1), carnitine palmitoyl transferase 1 (CPT-1A), hydroxymethylglutaryl-CoA synthase 2 (HMGCS2), lipoprotein lipase (LPL), branched-chain amino acid transferase (BCAT1) (I). Amino acid depletion in liver was determined by immunodetection of initiation factor 2α and its phosphorylated form (K). Male mice were used for the experiments.

It has been reported that FGF21 causes browning of white adipose tissue [44,45]. In agreement with this notion, ad libitum fed 6 months old *Slc6a19 (−/−)* mice had significantly elevated levels of FGF21 (Figure 5A), which were also observed at a slightly lower level in 2 months old mice (Figure 5B). In fasting animals, which generally have high FGF21 levels, only a small increase was observed (Figure S3A). Blood metabolites were also found to be consistent with elevated levels of FGF21 (Figure 5C–H) [44]. Ketone bodies and creatinine were significantly increased, blood glucose was normal, while serum triglyceride, cholesterol and non-esterified fatty acid levels were reduced. Under fasting conditions only small differences of metabolite levels were observed (Figure S3B–D). Consistent with this metabolite profile we found increased expression of fgf21 in the liver (Figure 5I). The fgf21 gene is regulated by the heteromeric transcription factor PPARa/RXR in liver [46]. However, in microarray studies we did not find increased expression of key PPARa target genes such as acyl-CoA oxidase (*ACOX1*) or carnitine palmitoyl transferase 1a (CPT1a) [47], which was confirmed by real time PCR (Figure 5I). Alternatively, amino acid starvation can trigger transcription of fgf21 through an amino acid response element in liver [17,18]. Consistent with reduced amino acid flux to the liver, we found elevated levels p-eIF2α in liver, similar to observations in the intestine reported by us previously (Figure 5K) [32].

### 3.5 *Slc6a19 (−/−) mice* have reduced mTOR signalling

**Figure 6:**
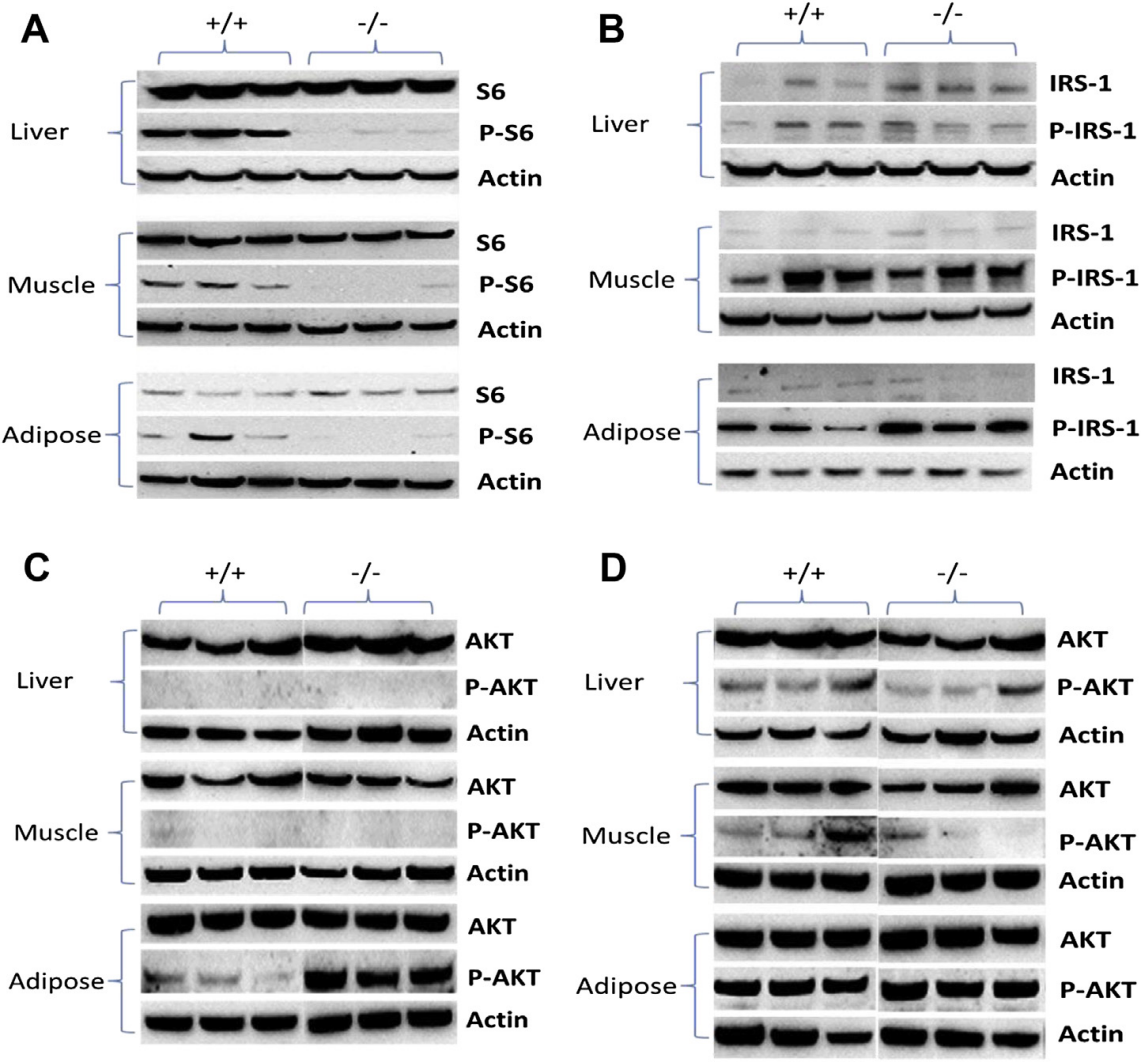
**Amino acid and insulin signalling in ad libitum fed wildtype and Slc6a19(−/−) mice**. (A) Signalling through the mTOR pathway was measured by analysing unphosphorylated (S6) and phosphorylated (P-S6) ribosomal protein S6 in liver, muscle and white adipose tissue. (B) Insulin signalling and insulin sensitivity was measured by analysing total and phosphorylated IRS-1 (P307-IRS-1) in liver, muscle and adipose tissue. (C) Detection of AKT and P473-AKT in liver, muscle and adipose tissue. (D) Detection of AKT and P473-AKT in liver, muscle and adipose tissue 30 min after 0.75 U/kg insulin injection. Male mice were used for the experiments.

FGF21 has been proposed to increase glucose removal by a variety of mechanisms [44,48,49], but glucose tolerance and insulin sensitivity could also be improved due to reduced amino acid availability. Elevated levels of serum amino acids will increase signalling through the mTOR pathway including p70S6 kinase. Activation of the mTOR pathway results in phosphorylation of several residues of the IRS-1 protein, which can cause insulin resistance [50,51]. Although the direct phosphorylation sites targeted by mTOR and p70S6 kinase are disputed [52], inhibition of mTOR by rapamycin significantly reduces phosphorylation of IRS-1 residues S302, S307 and S318 [51]. Phosphorylation of these sites is associated with insulin resistance and also results in IRS-1 degradation. In our experiments we used ribosomal protein S6 phosphorylation as a readout for mTOR and p70S6 kinase activity and monitored insulin receptor sensitivity by detection of IRS-1(pS307) and protein kinase Akt(pS473) phosphorylation. Consistent with lower amino acid availability, mTOR signalling was found to be reduced in muscle, adipose and liver from ad libitum fed animals (Figure 6A). No difference was observed in fasting animals (Figure S3E). Levels of IRS-1 were higher in the liver of *Slc6a19 (−/−)* mice, while adipose and muscle showed little difference (Figure 6B). If anything, levels of IRS-1(pS307) were increased in muscle and adipose tissue (Figure 6B). Levels of protein kinase Akt were similar in liver, muscle and adipose tissue in both groups of mice. pAkt(S473) was not detectable in unstimulated liver and muscle, but was easily detected in adipose tissue of *Slc6a19 (−/−)* mice (Figure 6C). After stimulation with insulin, similar levels of pAKT were detected in both groups of mice in all three tissues (Figure 6D).

### 3.6 *Slc6a19 (−/−) mice* show reduced hepatic glucose output and liver triglycerides

**Figure 7:**
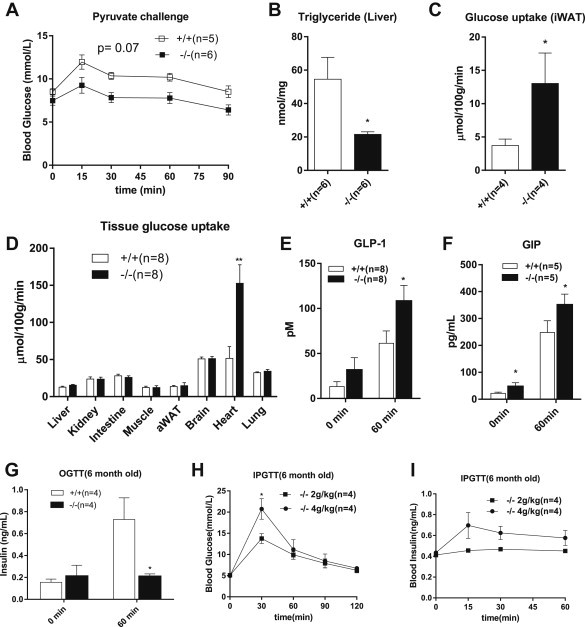
**Regulation of glucose metabolism in wildtype and Slc6a19(−/−) mice**. (A) Pyruvate (2 g/kg) was injected IP to measure hepatic glucose output in 6 months old fasting mice; (B) liver content of triglycerides. (C, D) Glucose (2 mg/kg) mixed with [^14^C]deoxyglucose was injected IP to measure glucose uptake in a variety of tissues after 40 min. Serum levels of GLP-1 (E) and GIP (F) were determined before and 60 min after oral food consumption. Insulin levels were measured before and 60 min after glucose gavage (G). Blood glucose (H) and insulin levels (I) were measured after IP injection of glucose (2 g and 4 g/kg) into Slc6a19 (−/−) mice. Male mice were used for the experiments.

Hepatic insulin resistance is correlated with enhanced glucose output via gluconeogenesis and elevated liver triglycerides. To investigate the effect of B^0^AT1 deficiency on liver gluconeogenesis we used a pyruvate challenge. The data show a slightly reduced glucose output after IP injection of pyruvate (Figure 7A), with borderline significance when the starting glucose concentration was normalised (AUC normalised, p = 0.07). Moreover, we also found reduced levels of triglycerides (Figure 7B), which may result from conversion of liver fat into ketone bodies.

### 3.7 *Slc6a19 (−/−) mice* show increased levels of GLP-1

As shown above, UCP-1 was upregulated in inguinal white adipose tissue, which was accompanied by a significant increase in glucose consumption (Figure 7C). To identify other organs which contributed to glucose removal, we injected radiolabelled glucose during an IPGTT and measured its distribution among organs after 1 h (Figure 7D). No notable difference was observed in insulin-sensitive muscle and abdominal white adipose tissue. Glucose consumption in BAT (Figure 4D), intestine, brain and lung was also unaltered. Gluconeogenesis in liver and kidney may confound the absolute values in these tissues, but overall no difference was observed. By contrast, glucose consumption by the heart was significantly increased in *Slc6a19 (−/−)* mice. It has been reported that GLP-1 increases glucose consumption in heart [53,54]. Since amino acids prevail in the lumen of the intestine for longer periods and are found in further distal sections than in wildtype mice (Figure 3D), we hypothesised that incretin secretion could be increased. In agreement with this notion we found that GLP-1 and GIP levels were significantly elevated in *Slc6a19* (−/−) mice after oral food consumption (Figure 7E,F). Surprisingly, Slc6a19 (−/−) have lower insulin secretion than wildtype mice even after glucose gavage (Figure 7G), suggesting that the incretin effect is subdued. To investigate whether insulin secretion was principally defective, we doubled the amount of glucose injected during an IPGTT (4 g/kg). In this experiment the blood glucose response in *Slc6a19 (*−/−*)* mice became similar to that of the wildtype at 2 g/kg glucose injection (Figure 7H) and insulin secretion was observed (Figure 7I). Thus insulin secretion is not defective, but the threshold appears to be altered. We wondered whether B^0^AT1 could directly influence amino acid-induced insulin release as its mRNA has been detected in small amounts in pancreas [32]. However, several attempts to identify B^0^AT1 protein expression in the pancreas using antibodies previously used to detect B^0^AT1 protein in kidney and intestine [32] failed to provide evidence for expression in β-cells (data not shown).

In summary, our results suggest that the metabolic phenotype of *Slc6a19 (−/−)* mice can be explained by the heart acting as a glucose sink, most likely caused by increased GLP-1 secretion from the intestine. Due to the reduced flux of amino acids to the liver, FGF21 is produced, which causes up-regulation of UCP-1 in subcutaneous fat. Both mechanisms will remove glucose without requiring insulin. FGF21 in addition causes break-down of fat to produce ketone bodies.

## 4 Discussion

Lack of neutral amino absorption in the intestine caused by B^0^AT1 deficiency results in two opposing signals that contribute to the metabolic phenotype of *Slc6a19* nullizygous mice. First, reduced uptake in the intestine causes accumulation of amino acids in the lumen and occurrence of free amino acids in more distal parts of the intestine. This phenomenon has also been observed in ACE2-deficient mice, which lack expression of B^0^AT1 in the intestine because of the missing trafficking protein that brings B^0^AT1 to the cell surface [55]. Further indication of distal occurrence of amino acids is the production of short-chain fatty acids resulting from fermentation by the intestinal microflora. Overall the high luminal amino acid concentration sends a “full” signal to the body, particularly through the secretion of GLP-1 and GIP. Both peptide hormones are secreted by specific enteroendocrine cells, which sense luminal amino acid content as a signal for secretion [56,57]. The second signal is generated by the liver. As evidenced by reduced mTOR signalling and phosphorylation of eIF2α, lack of B^0^AT1 in the intestine reduces the influx of amino acids through the portal vein to the liver after a meal. This amino acid starvation response is known to increase *fgf21* transcription through amino acid response elements in the promoter region of the gene [17,18]. Surprisingly, the difference of FGF21 serum levels (27-fold) was much higher than the change of mRNA expression in liver (5-fold). These data were confirmed in microarray experiments showing a 6-fold increase in liver, while FGF21 expression was below threshold in adipose tissue and muscle (data not shown). Western blot of pancreas tissue also revealed no difference in FGF21 levels between the two groups of mice (data not shown). These results point to a possible increase of the biological half-life in *Slc6a19 (−/−)* mice, which remains to be determined. The phenotype of *Slc6a19 (−/−)* mice appears to be a combination of GLP-1 and FGF-21 induced signals. Reduced hepatic glucose output, increased oxidation of fatty acids and ketogenesis are metabolic programs elicited by FGF21 through the FGF21/β-klotho receptor [20]. Moreover, reduced insulin levels have also been reported after injection of FGF21 [58]. The response to FGF21 secretion in *Slc6a19 (−/−)* mice is similar to that in other model systems [44,59]. In view of adipose tissue being the major target of FGF21 action [21], it is tempting to speculate that FGF21 may be involved in phosphorylation of AKT in non-insulin treated adipose tissue of *Slc6a19 (−/−)* mice. It has furthermore been shown that FGF21 increases adiponectin expression and excretion in adipose tissue and that this could be the basis for many of its metabolic effects [58]. In microarrays we could not detect enhanced expression of adiponectin in abdominal adipose tissue, but it remains to be shown whether it is up-regulated in inguinal WAT.

Increased glucose consumption by the heart is thought to arise through GLP-1 [53]. Together both signals result in a phenotype that is highly desirable in the context of managing metabolic syndrome and diabetes.

The increased glucose consumption by the heart (1 μmol/g*min) makes a significant contribution to the IPGTT. The average weight of a mouse heart is 150 mg resulting in an additional glucose consumption of 150 nmol/min. In the standard IPGTT (Figure 1) we observed a difference between wildtype and *Slc6a19 (*−/−*)* mice of about 8 mM/30 min. Assuming a blood volume of 1.5 ml, this amounts to 400 nmol glucose/min, explaining about 40% of the time course. The reminder most likely can be attributed to glucose consumption by subcutaneous WAT and reduced glucose output by the liver. Over time adipose tissue and triglycerides in liver are reduced due to the conversion into ketone bodies.

Why β-cells release so little insulin despite increased secretion of insulinotropic peptide hormones GLP-1 and GIP remains to be determined. In part, it is caused by largely insulin-independent glucose removal by the heart and inguinal adipose tissue. As a result blood, glucose levels rise more slowly, thereby reducing insulin secretion. However, at a plasma glucose concentration of >10 mM, as observed during the IPGTT, significant insulin secretion is expected. A similar lack of response has also been observed in ACE2 (−/−) mice, which lack B^0^AT1 in the intestine [60,61]. In terms of managing diabetes and metabolic syndrome, this reduced response may offer additional benefits, such as protection of β-cells against endoplasmic reticulum stress [62]. In addition, it establishes amino acid absorption as a significant modulator of fuel-induced insulin secretion. In humans and rats GLP-1 and GIP send satiation signals to the brain, which we do not seem to observe, for *Slc6a19 (−/−)* mice appear to be slightly hyperphagic, when normalised to body weight.

The lack of B^0^AT1 in the kidney is likely to contribute to the observed phenotype, as suggested by a similar metabolic phenotype observed in collectrin deficient animals [63], which lack B^0^AT1 only in the kidney. ACE2 (−/−) mice, which lack B^0^AT1 in the intestine, have a distinct phenotype caused by increased levels of angiotensin II [61], but share reduced insulin secretion.

Despite the loss of amino acids in the urine and reduced uptake in the intestine, plasma amino acid levels in fasting animals remained largely normal. Two factors contributed to this surprising result. First, amino acids were spared as fuels as demonstrated by the reduced urea concentration in blood plasma and urine. Secondly, muscle protein turnover and degradation was reduced as indicated by lower 3-methylhistidine concentrations in blood plasma. This result is consistent with the observation that increased plasma concentrations of branched-chain amino acids cause increased protein turnover and energy consumption [64].

## 5 Conclusions

Slc6a19 (−/−) mice demonstrate a significant role of neutral amino acids in the regulation of fuel metabolism, which has only partially appreciated previously. Curtailing amino acid absorption in the intestine causes increased production of GLP-1 by the intestine and increases FGF-21 production by the liver. These results establish a novel target to treat type 2 diabetes and its associated comorbidities.

## Author contributions

Y.J. Performed experiments and analysed data, A.J.R. designed, conducted and analysed experiments and edited the manuscript, S.H. discussed data and edited the manuscript, T.P.S. performed experiments and analysed data, A.B. performed experiments, A.P. performed experiments and analysed data, D.S. discussed data and edited the manuscript, S.B. designed the study, discussed all aspects of the work and wrote the manuscript.

## References

[1] Tremblay F., Lavigne C., Jacques H., Marette A. (2007). Role of dietary proteins and amino acids in the pathogenesis of insulin resistance. Annual Review of Nutrition.

[2] Krebs M., Krssak M., Bernroider E., Anderwald C., Brehm A., Meyerspeer M. (2002). Mechanism of amino acid-induced skeletal muscle insulin resistance in humans. Diabetes.

[3] Tessari P., Inchiostro S., Biolo G., Duner E., Nosadini R., Tiengo A. (1985). Hyperaminoacidaemia reduces insulin-mediated glucose disposal in healthy man. Diabetologia.

[4] Shah O.J., Wang Z., Hunter T. (2004). Inappropriate activation of the TSC/Rheb/mTOR/S6K cassette induces IRS1/2 depletion, insulin resistance, and cell survival deficiencies. Current Biology.

[5] Copps K.D., White M.F. (2012). Regulation of insulin sensitivity by serine/threonine phosphorylation of insulin receptor substrate proteins IRS1 and IRS2. Diabetologia.

[6] Macotela Y., Emanuelli B., Bang A.M., Espinoza D.O., Boucher J., Beebe K. (2011). Dietary leucine–an environmental modifier of insulin resistance acting on multiple levels of metabolism. PLoS One.

[7] Zhang Y., Guo K., LeBlanc R.E., Loh D., Schwartz G.J., Yu Y.H. (2007). Increasing dietary leucine intake reduces diet-induced obesity and improves glucose and cholesterol metabolism in mice via multimechanisms. Diabetes.

[8] Bernard J.R., Liao Y.H., Hara D., Ding Z., Chen C.Y., Nelson J.L. (2011). An amino acid mixture improves glucose tolerance and insulin signaling in Sprague-Dawley rats. American Journal of Physiology. Endocrinology and Metabolism.

[9] Xiao F., Huang Z., Li H., Yu J., Wang C., Chen S. (2011). Leucine deprivation increases hepatic insulin sensitivity via GCN2/mTOR/S6K1 and AMPK pathways. Diabetes.

[10] Lu J., Xie G., Jia W. (2013). Insulin resistance and the metabolism of branched-chain amino acids. Frontier Medical.

[11] Solon-Biet S.M., McMahon A.C., Ballard J.W., Ruohonen K., Wu L.E., Cogger V.C. (2014). The ratio of macronutrients, not caloric intake, dictates cardiometabolic health, aging, and longevity in ad libitum-fed mice. Cell Metabolism.

[12] Newgard C.B., An J., Bain J.R., Muehlbauer M.J., Stevens R.D., Lien L.F. (2009). A branched-chain amino acid-related metabolic signature that differentiates obese and lean humans and contributes to insulin resistance. Cell Metabolism.

[13] Wang T.J., Larson M.G., Vasan R.S., Cheng S., Rhee E.P., McCabe E. (2011). Metabolite profiles and the risk of developing diabetes. Nature Medicine.

[14] Newgard C.B. (2012). Interplay between lipids and branched-chain amino acids in development of insulin resistance. Cell Metabolism.

[15] Kilberg M.S., Shan J., Su N. (2009). ATF4-dependent transcription mediates signaling of amino acid limitation. Trends in Endocrinology & Metabolism.

[16] Krokowski D., Han J., Saikia M., Majumder M., Yuan C.L., Guan B.J. (2013). A self-defeating anabolic program leads to beta-cell apoptosis in endoplasmic reticulum stress-induced diabetes via regulation of amino acid flux. The Journal of Biological Chemistry.

[17] Laeger T., Henagan T.M., Albarado D.C., Redman L.M., Bray G.A., Noland R.C. (2014). FGF21 is an endocrine signal of protein restriction. The Journal of Clinical Investigation.

[18] De Sousa-Coelho A.L., Marrero P.F., Haro D. (2012). Activating transcription factor 4-dependent induction of FGF21 during amino acid deprivation. The Biochemical Journal.

[19] Kharitonenkov A., Adams A.C. (2014). Inventing new medicines: the FGF21 story. Molecular Metabolism.

[20] Owen B.M., Mangelsdorf D.J., Kliewer S.A. (2015). Tissue-specific actions of the metabolic hormones FGF15/19 and FGF21. Trends in Endocrinology and Metabolism: TEM.

[21] Adams A.C., Yang C., Coskun T., Cheng C.C., Gimeno R.E., Luo Y. (2012). The breadth of FGF21's metabolic actions are governed by FGFR1 in adipose tissue. Molecular Metabolism.

[22] Holland W.L., Adams A.C., Brozinick J.T., Bui H.H., Miyauchi Y., Kusminski C.M. (2013). An FGF21-adiponectin-ceramide axis controls energy expenditure and insulin action in mice. Cell Metabolism.

[23] Reimann F., Williams L., da Silva Xavier G., Rutter G.A., Gribble F.M. (2004). Glutamine potently stimulates glucagon-like peptide-1 secretion from GLUTag cells. Diabetologia.

[24] Thomas F.B., Mazzaferri E.L., Crockett S.E., Mekhjian H.S., Gruemer H.D., Cataland S. (1976). Stimulation of secretion of gastric inhibitory polypeptide and insulin by intraduodenal amino acid perfusion. Gastroenterology.

[25] Westerterp-Plantenga M.S., Lemmens S.G., Westerterp K.R. (2012). Dietary protein - its role in satiety, energetics, weight loss and health. The British Journal of Nutrition.

[26] Simpson S.J., Raubenheimer D. (2005). Obesity: the protein leverage hypothesis. Obesity Reviews.

[27] Journel M., Chaumontet C., Darcel N., Fromentin G., Tome D. (2012). Brain responses to high-protein diets. Advances in Nutrition.

[28] Houde V.P., Brule S., Festuccia W.T., Blanchard P.G., Bellmann K., Deshaies Y. (2010). Chronic rapamycin treatment causes glucose intolerance and hyperlipidemia by upregulating hepatic gluconeogenesis and impairing lipid deposition in adipose tissue. Diabetes.

[29] Felig P. (1975). Amino acid metabolism in man. The Annual Review of Biochemistry.

[30] Broer S. (2008). Amino acid transport across mammalian intestinal and renal epithelia. Physiological Reviews.

[31] Broer A., Klingel K., Kowalczuk S., Rasko J.E., Cavanaugh J., Broer S. (2004). Molecular cloning of mouse amino acid transport system B0, a neutral amino acid transporter related to Hartnup disorder. The Journal of Biological Chemistry.

[32] Broer A., Juelich T., Vanslambrouck J.M., Tietze N., Solomon P.S., Holst J. (2011). Impaired nutrient signaling and body weight control in a Na+ neutral amino acid cotransporter (Slc6a19)-deficient mouse. The Journal of Biological Chemistry.

[33] Nassl A.M., Rubio-Aliaga I., Fenselau H., Marth M.K., Kottra G., Daniel H. (2011). Amino acid absorption and homeostasis in mice lacking the intestinal peptide transporter PEPT1. The American Journal of Physiology-Gastrointestinal and Liver Physiology.

[34] Broer S. (2009). The role of the neutral amino acid transporter B0AT1 (SLC6A19) in Hartnup disorder and protein nutrition. IUBMB Life.

[35] Seow H.F., Broer S., Broer A., Bailey C.G., Potter S.J., Cavanaugh J.A. (2004). Hartnup disorder is caused by mutations in the gene encoding the neutral amino acid transporter SLC6A19. Nature Genetics.

[36] Kleta R., Romeo E., Ristic Z., Ohura T., Stuart C., Arcos-Burgos M. (2004). Mutations in SLC6A19, encoding B0AT1, cause Hartnup disorder. Nature Genetics.

[37] Danilczyk U., Sarao R., Remy C., Benabbas C., Stange G., Richter A. (2006). Essential role for collectrin in renal amino acid transport. Nature.

[38] Kowalczuk S., Broer A., Tietze N., Vanslambrouck J.M., Rasko J.E., Broer S. (2008). A protein complex in the brush-border membrane explains a Hartnup disorder allele. Faseb Journal.

[39] Tschop M.H., Speakman J.R., Arch J.R., Auwerx J., Bruning J.C., Chan L. (2012). A guide to analysis of mouse energy metabolism. Nature Methods.

[40] Speakman J.R., Fletcher Q., Vaanholt L. (2013). The ‘39 steps’: an algorithm for performing statistical analysis of data on energy intake and expenditure. Disease Models & Mechanisms.

[41] Carpenter A.E., Jones T.R., Lamprecht M.R., Clarke C., Kang I.H., Friman O. (2006). CellProfiler: image analysis software for identifying and quantifying cell phenotypes. Genome Biology.

[42] Levy L.L., Scriver C.R., Beaudet A.L., Sly W.S., Valle D. (2001). Hartnup disorder. The metabolic & molecular bases of inherited diseases.

[43] Speakman J.R. (2013). Measuring energy metabolism in the mouse - theoretical, practical, and analytical considerations. Frontiers in Physiology.

[44] Kharitonenkov A., Shiyanova T.L., Koester A., Ford A.M., Micanovic R., Galbreath E.J. (2005). FGF-21 as a novel metabolic regulator. The Journal of Clinical Investigation.

[45] Coskun T., Bina H.A., Schneider M.A., Dunbar J.D., Hu C.C., Chen Y. (2008). Fibroblast growth factor 21 corrects obesity in mice. Endocrinology.

[46] Iglesias P., Selgas R., Romero S., Diez J.J. (2012). Biological role, clinical significance, and therapeutic possibilities of the recently discovered metabolic hormone fibroblastic growth factor 21. European Journal of Endocrinology.

[47] McMullen P.D., Bhattacharya S., Woods C.G., Sun B., Yarborough K., Ross S.M. (2014). A map of the PPARalpha transcription regulatory network for primary human hepatocytes. Chemico-Biological Interactions.

[48] Mashili F.L., Austin R.L., Deshmukh A.S., Fritz T., Caidahl K., Bergdahl K. (2011). Direct effects of FGF21 on glucose uptake in human skeletal muscle: implications for type 2 diabetes and obesity. Diabetes/Metabolism Research and Reviews.

[49] Markan K.R., Naber M.C., Ameka M.K., Anderegg M.D., Mangelsdorf D.J., Kliewer S.A. (2014). Circulating FGF21 is liver derived and enhances glucose uptake during refeeding and overfeeding. Diabetes.

[50] Aguirre V., Werner E.D., Giraud J., Lee Y.H., Shoelson S.E., White M.F. (2002). Phosphorylation of Ser307 in insulin receptor substrate-1 blocks interactions with the insulin receptor and inhibits insulin action. The Journal of Biological Chemistry.

[51] Hancer N.J., Qiu W., Cherella C., Li Y., Copps K.D., White M.F. (2014). Insulin and metabolic stress stimulate multisite serine/threonine phosphorylation of insulin receptor substrate 1 and inhibit tyrosine phosphorylation. The Journal of Biological Chemistry.

[52] Rajan M.R., Fagerholm S., Jonsson C., Kjolhede P., Turkina M.V., Stralfors P. (2013). Phosphorylation of IRS1 at serine 307 in response to insulin in human adipocytes is not likely to be catalyzed by p70 ribosomal S6 kinase. PLoS One.

[53] Nikolaidis L.A., Elahi D., Shen Y.T., Shannon R.P. (2005). Active metabolite of GLP-1 mediates myocardial glucose uptake and improves left ventricular performance in conscious dogs with dilated cardiomyopathy. American Journal of Physiology – Heart and Circulatory Physiology.

[54] Giannocco G., Oliveira K.C., Crajoinas R.O., Venturini G., Salles T.A., Fonseca-Alaniz M.H. (2013). Dipeptidyl peptidase IV inhibition upregulates GLUT4 translocation and expression in heart and skeletal muscle of spontaneously hypertensive rats. European Journal of Pharmacology.

[55] Singer D., Camargo S.M., Ramadan T., Schafer M., Mariotta L., Herzog B. (2012). Defective intestinal amino acid absorption in Ace2 null mice. The American Journal of Physiology-Gastrointestinal and Liver Physiology.

[56] Young S.H., Rey O., Sternini C., Rozengurt E. (2010). Amino acid sensing by enteroendocrine STC-1 cells: role of the Na+-coupled neutral amino acid transporter 2. American Journal of Physiology. Cell Physiology.

[57] Lindgren O., Pacini G., Tura A., Holst J.J., Deacon C.F., Ahren B. (2014). Incretin effect after oral amino acid ingestion in humans. The Journal of Clinical Endocrinology and Metabolism.

[58] Lin Z., Tian H., Lam K.S., Lin S., Hoo R.C., Konishi M. (2013). Adiponectin mediates the metabolic effects of FGF21 on glucose homeostasis and insulin sensitivity in mice. Cell Metabolism.

[59] Badman M.K., Pissios P., Kennedy A.R., Koukos G., Flier J.S., Maratos-Flier E. (2007). Hepatic fibroblast growth factor 21 is regulated by PPARalpha and is a key mediator of hepatic lipid metabolism in ketotic states. Cell Metabolism.

[60] Niu M.J., Yang J.K., Lin S.S., Ji X.J., Guo L.M. (2008). Loss of angiotensin-converting enzyme 2 leads to impaired glucose homeostasis in mice. Endocrine.

[61] Bernardi S., Tikellis C., Candido R., Tsorotes D., Pickering R.J., Bossi F. (2015). ACE2 deficiency shifts energy metabolism towards glucose utilization. Metabolism.

[62] Biden T.J., Boslem E., Chu K.Y., Sue N. (2014). Lipotoxic endoplasmic reticulum stress, beta cell failure, and type 2 diabetes mellitus. Trends in Endocrinology and Metabolism: TEM.

[63] Malakauskas S.M., Kourany W.M., Zhang X.Y., Lu D., Stevens R.D., Koves T.R. (2009). Increased insulin sensitivity in mice lacking collectrin, a downstream target of HNF-1alpha. Molecular Endocrinology.

[64] She P., Reid T.M., Bronson S.K., Vary T.C., Hajnal A., Lynch C.J. (2007). Disruption of BCATm in mice leads to increased energy expenditure associated with the activation of a futile protein turnover cycle. Cell Metabolism.

