# Supplementary material for: Mice lacking neutral amino acid transporter B0AT1 (Slc6a19) have elevated levels of FGF21 and GLP-1 and improved glycaemic control
Source: Mol Metab. 2015 Feb 16;4(5):406–17. doi: 10.1016/j.molmet.2015.02.003 (PMC4421019; doi:10.1016/j.molmet.2015.02.003)
Supplement: Supplementary file 1 [file mmc1.pptx]

## Slide 1
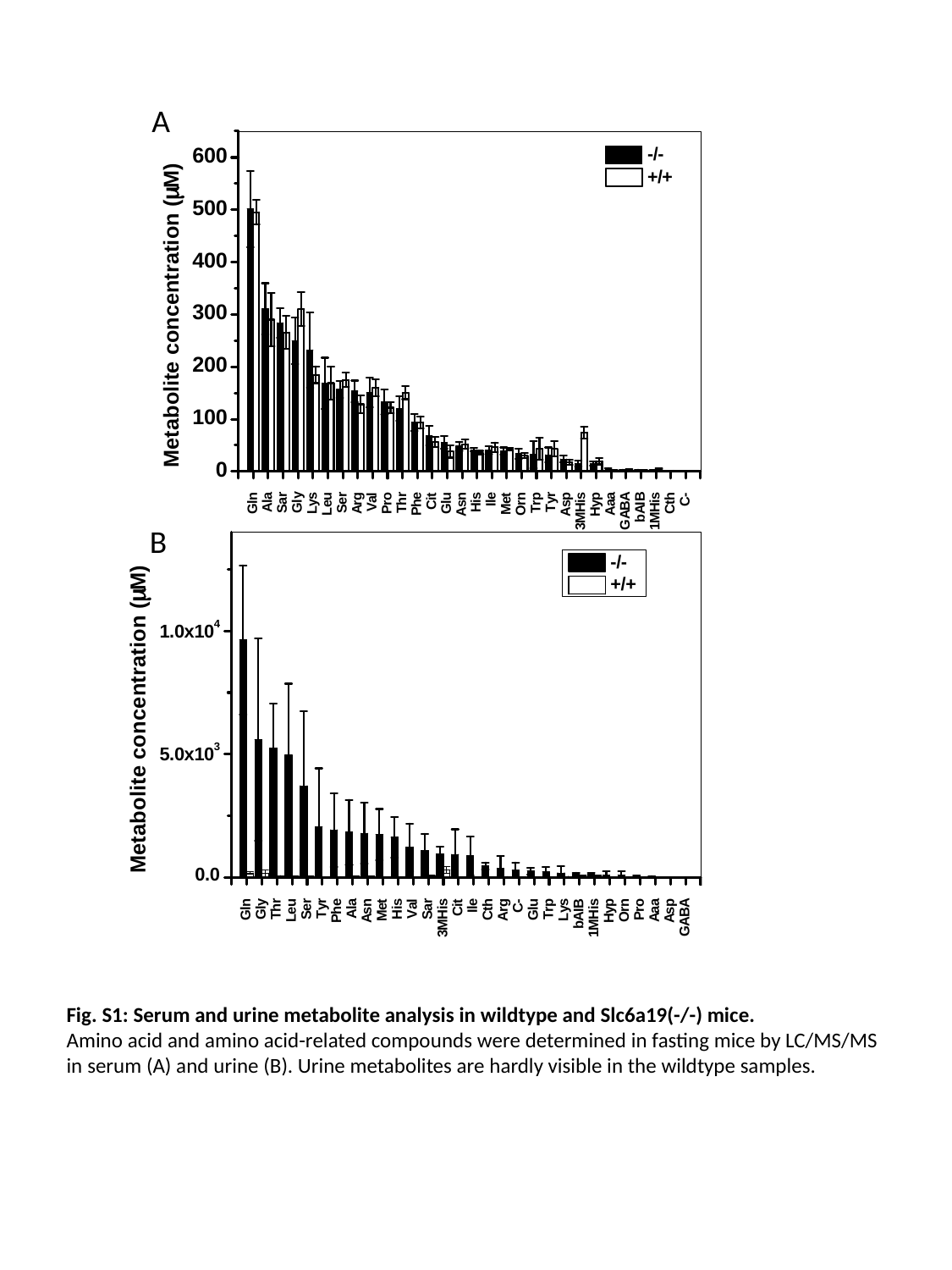

A
B
Fig. S1: Serum and urine metabolite analysis in wildtype and Slc6a19(-/-) mice.
Amino acid and amino acid-related compounds were determined in fasting mice by LC/MS/MS in serum (A) and urine (B). Urine metabolites are hardly visible in the wildtype samples.

## Slide 2
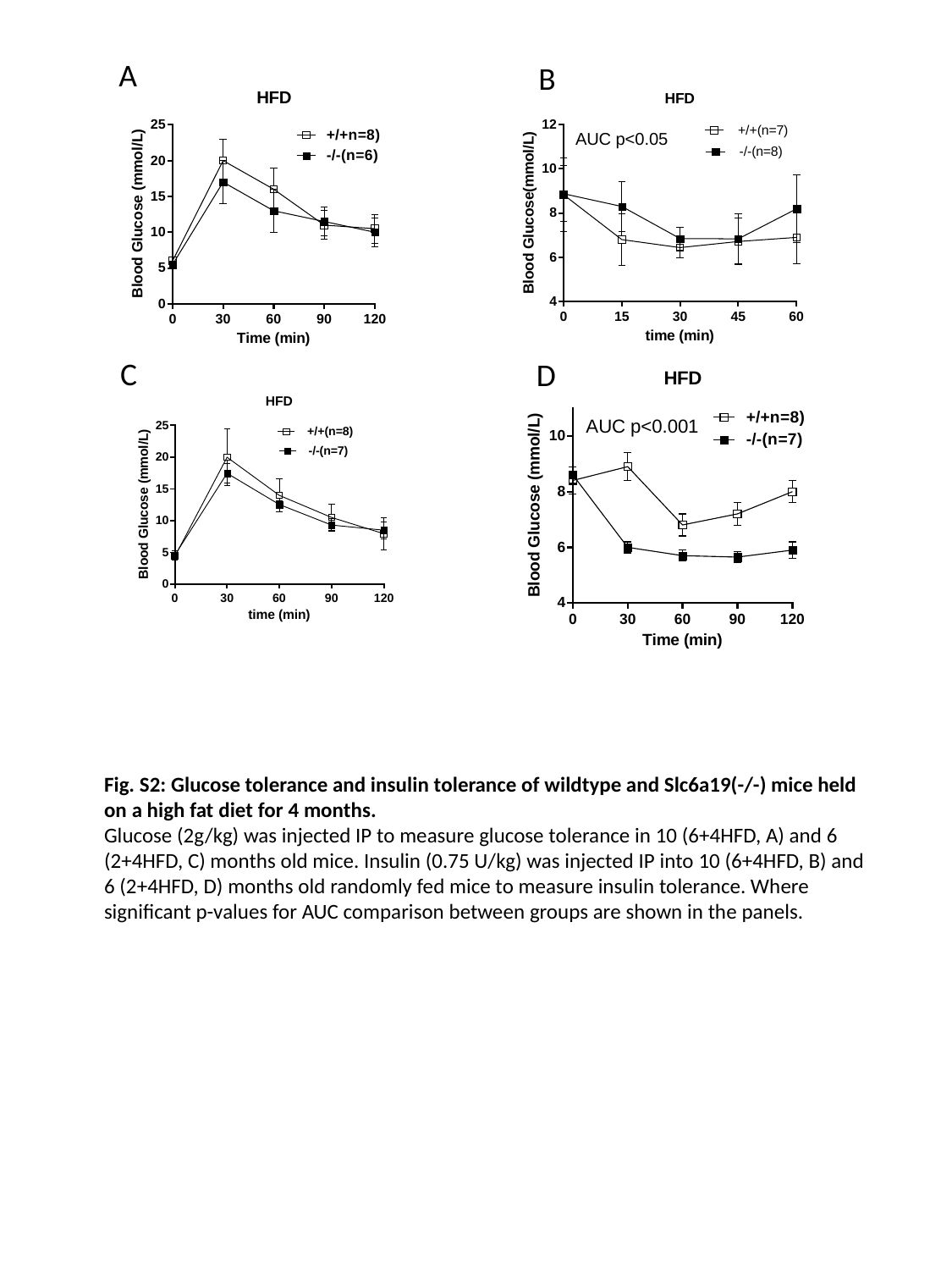

A
B
C
D
Fig. S2: Glucose tolerance and insulin tolerance of wildtype and Slc6a19(-/-) mice held on a high fat diet for 4 months.
Glucose (2g/kg) was injected IP to measure glucose tolerance in 10 (6+4HFD, A) and 6 (2+4HFD, C) months old mice. Insulin (0.75 U/kg) was injected IP into 10 (6+4HFD, B) and 6 (2+4HFD, D) months old randomly fed mice to measure insulin tolerance. Where significant p-values for AUC comparison between groups are shown in the panels.

## Slide 3
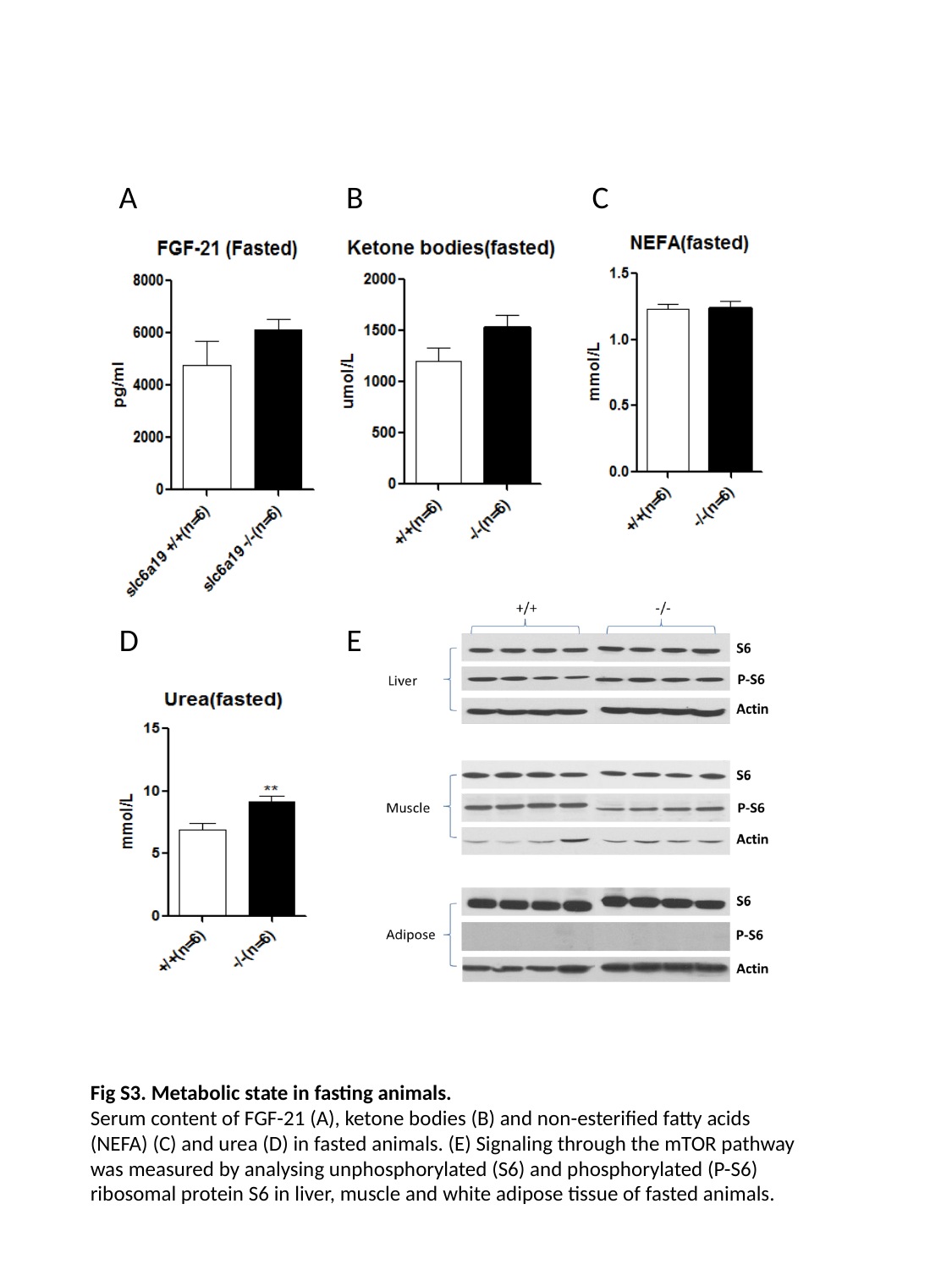

B
C
A
D
E
Fig S3. Metabolic state in fasting animals.
Serum content of FGF-21 (A), ketone bodies (B) and non-esterified fatty acids (NEFA) (C) and urea (D) in fasted animals. (E) Signaling through the mTOR pathway was measured by analysing unphosphorylated (S6) and phosphorylated (P-S6) ribosomal protein S6 in liver, muscle and white adipose tissue of fasted animals.
