# Supplementary material for: Mice lacking neutral amino acid transporter B0AT1 (Slc6a19) have elevated levels of FGF21 and GLP-1 and improved glycaemic control
Source: Mol Metab. 2015 Feb 16;4(5):406–17. doi: 10.1016/j.molmet.2015.02.003 (PMC4421019; doi:10.1016/j.molmet.2015.02.003)
Supplement: Supplementary file 2 [file mmc2.docx]

Supplementary Table 1: Assays used for metabolic analysis

| Compound | Assay | Manufacturer |
| --- | --- | --- |
| Ketone bodies | Autokit Total Ketone Bodies | WAKO |
| Non-esterified fatty acid | NEFA-HR | WAKO |
| Triglyceride | TR-0100-1KT | Sigma-Aldrich |
| Urea | Urea Assay Kit | Biochain |
| Cholesterol | CH200 | Randox |
| FGF-21 | Mouse/Rat FGF-21 | R&D Systems |
| Glucagon like peptide-1 | Multi Species GLP-1 Total ELISA, EZGLP1T-36K | Merck Millipore |
| Gastric inhibitory polypeptide | Rat/Mouse GIP (total) ELISA | Merck Millipore |
| Insulin | Ultrasensitive mouse insulin ELISA | ALPCO |

Supplementary Table 2: Antibodies used in this study

| **Target** | **Primary** | **Secondary** |
| --- | --- | --- |
| β-Actin | 1:2000 (WB), rabbit polyclonal (Abcam) | 1:2000 donkey anti rabbit IgG, HRP conjugated (GE Healthcare) |
| S6 | 1:1000 (WB), rabbit polyclonal (Cell Signaling) | 1:1000 donkey anti rabbit IgG, HRP conjugated (GE Healthcare) |
| Phospho-S6 | 1:1000 (WB), rabbit polyclonal (Cell Signaling) | 1:1000 donkey anti rabbit IgG, HRP conjugated (GE Healthcare) |
| Irs-1 | 1:1000 (WB), rabbit polyclonal (Cell Signaling) | 1:1000 donkey anti rabbit IgG, HRP conjugated (GE Healthcare |
| Phospho-Irs-1 | 1:1000(WB), rabbit polyclonal (Pierce) | 1:1000 donkey anti rabbit IgG, HRP conjugated (GE Healthcare) |
| Ucp-1 | 1:1000(WB), rabbit polyclonal (Calbiochem) | 1:1000 donkey anti rabbit IgG, HRP conjugated (GE Healthcare) |
| eIF2α | 1:1000(WB), rabbit polyclonal (Cell Signaling) | 1:1000 donkey anti rabbit IgG, HRP conjugated (GE Healthcare) |
| Phospho eIF2α | 1:1000(WB), rabbit polyclonal (Cell Signaling) | 1:1000 donkey anti rabbit IgG, HRP conjugated (GE Healthcare) |
| Akt(Total) | 1:1000(WB), rabbit polyclonal (Abcam) | 1:1000 donkey anti rabbit IgG, HRP conjugated (GE Healthcare) |
| Phospho Akt(S473) | 1:1000(WB), rabbit polyclonal (Cell Signaling) | 1:1000 donkey anti rabbit IgG, HRP conjugated (GE Healthcare) |
